# Saccharification and hydrolytic enzyme production of alkali pre-treated wheat bran by *Trichoderma virens* under solid state fermentation

**DOI:** 10.1186/s12896-015-0158-4

**Published:** 2015-05-28

**Authors:** Reda M. El-Shishtawy, Saleh A. Mohamed, Abdullah M. Asiri, Abu-bakr M. Gomaa, Ibrahim H. Ibrahim, Hasan A. Al-Talhi

**Affiliations:** Chemistry Department, Faculty of Science, King Abdulaziz University, P.O. Box 80203, Jeddah, 21589 Saudi Arabia; Biochemistry Department, Faculty of Science, King Abdulaziz University, Jeddah, Kingdom of Saudi Arabia; The Center of Excellence for Advanced Materials Research, King Abdulaziz University, Jeddah, 21589 Saudi Arabia; Biology Department, Faculty of Science, King Abdulaziz University, Jeddah, Kingdom of Saudi Arabia

**Keywords:** *Trichoderma* sp, Saccharification, Hydrolytic enzymes, Agriculture wastes

## Abstract

**Background:**

In continuation of our previously interest in the saccharification of agriculture wastes by *Bacillus megatherium* in solid state fermentation (SSF), we wish to report an investigation and comparative evaluation among *Trichoderma* sp. for the saccharification of four alkali-pretreated agricultural residues and production of hydrolytic enzymes, carboxymethyl cellulase (CMCase), filter paperase (FPase), pectinase (PGase) and xylanase (Xylase) in SSF. The optimization of the physiological conditions of production of hydrolytic enzymes and saccharification content from *Trichoderma virens* using alkali-pretreated wheat bran was the last goal.

**Methods:**

The physico-chemical parameters of SSF include incubation time, incubation temperature, moisture content of the substrate, incubation pH, supplementation with carbon and nitrogen sources were optimized.

**Results:**

Saccharification of different solid state fermentation sources wheat bran, date's seeds, grass and palm leaves, were tested for the production of fermentable sugar by *Trichoderma* sp. The maximum production of hydrolytic enzymes CMCase, FPase, PGase and Xylase and saccharification content were obtained on wheat bran. Time course, moisture content, optimum temperature, optimum pH, supplementation with carbon and nitrogen sources were optimized to achieve the maximum production of the hydrolytic enzymes, protein and total carbohydrate of *T. virens* using alkali pre-treated wheat bran. The maximum production of CMCase, FPase, PGase, Xylase, protein and carbohydrate content was recorded at 72 h of incubation, 50-70 % moisture, temperature 25-35 °C and pH 5. The influence of supplementary carbon and nitrogen sources was studied. While lactose and sucrose enhanced the activity of PGase from 79.2 to 582.9 and 632.6 U/g, starch inhibited all other enzymes. This was confirmed by maximum saccharification content. Among the nitrogen sources, yeast extract and urea enhanced the saccharification content and CMCase, PGase and Xylase.

**Conclusions:**

The results of this study indicated that alkali pre-treated wheat bran was a better substrate for saccharification and production of hydrolytic enzymes CMCase, FPase, PGase and xylase by *T. virens* compared to other alkali-pretreated agricultural residues tested.

## Background

Agricultural residues, forests and agro industrial practices generally accumulated in the environment and caused pollution problem. Active efforts were being made to convert these organic waste resources into either glucose or alcohol, and use this either as fuel or as a valuable starting material for chemical synthesis [1]. Saccharification of polysaccharides to glucose by microbial hydrolytic enzymes which had attracted the attention of the researchers, as this was the first step of bioconversion of organic material into valuable products such as sugar, fine chemicals and biofuels [2]. As the cost of cellulosic substrates play the central role in determining the economy of the saccharification process, lot of emphasis had been given to the usage of low price substrates and therefore screening of the agricultural wastes for release of sugars as organic wastes from renewable forest and agricultural residues [3]. The saccharification of different agro-wastes had been reported by other workers employing enzymes from different organisms [4–6].

Recently, a significant interest raised in using solid state fermentation (SSF) instead of submerged fermentation (SmF). The advantages of SSF in comparison to traditional SmF were better yields, easier recovery of products, the absence of foam formation and smaller reactor volumes. Moreover, contamination risks were significantly reduced due to the low water contents and, consequently, the volume of effluents decreases [7]. Another very important advantage was that, it permits the use of agricultural and agro-industrial residues as substrates which were converted into products with high commercial value like secondary metabolites, organic acids, pesticides, aromatic compounds, fuels and enzymes [8]. Furthermore, the utilization of these compounds helps in solving pollution, which otherwise caused their disposal [9]. For enzyme production, the costs of these techniques were lower and the production higher than submerged cultures [10,11].

Structural properties of cellulose such as the degree of crystallinity, the degree of polymerization and the surface area, limit accessibility of substrate to enzyme and had been demonstrated [12] to affect the rate of enzymatic hydrolysis of cellulose. Pretreatment methods, which disrupted the highly-ordered cellulose structure and the lignin-carbohydrate complex, remove lignin, and increase the surface area accessible to enzymes, promoted the hydrolysis, and increased the rate and extent of hydrolysis of cellulose in various lignocellulosic residues. The enzymatic hydrolysis of cellulosic materials correlated with the level of cellulose crystallinity [13] complete enzymatic hydrolysis of the polysaccharides of lignocelluloses required a concerted action of a complex array of hydrolases including cellulase, xylanase, pectinase, and other side-group cleavage enzymes [14].

Several cell-decomposing microorganisms produce cellulases which were the most economic and available sources for fermentable sugar production, because these microorganisms could grow on inexpensive media. The genus *Trichoderma,* filamentous ascomycetes were widely used in industrial applications because of high secretary capacity and inducible promoting characteristics [15]. The structural complexity were often easily degraded by xylanases, mannanases etc. which were present in some cellulase preparations, so that their presence may actually lead to increased production of reducing sugars and greater susceptibility of the residual cellulose [16–18].

In continuation of our interest in the saccharification of agriculture wastes by SSF [19], we wished to report an investigation and comparative evaluation among *Trichoderma* sp., *T. reesei, T. viride, T. harzianum* and *T. virens* for the saccharification of four alkali-pretreated agricultural residues, wheat bran, date’s seeds, wild grass and palm’s leaves under solid state fermentation for the production of hydrolytic enzymes, carboxymethyl cellulase (CMCase), filter paperase (FPase), pectinase (PGase) and xylanase (Xylase). The polysaccharide composition of these agricultural residues included different concentrations from cellulose, hemicelluloses and lignin [20–22]. The optimization of the physiological conditions of production of hydrolytic enzymes and saccharification content from *T. virens* using alkali-pretreated wheat bran was the last goal.

## Methods

### Microorganisms

*T. reesei, T. viride, T. harzianum* and *T. virens* were obtained from National Research Centre, Cairo, Egypt and maintained on potato dextrose agar. The slants were grown at 28 °C for seven days and stored at 4 °C.

### Pretreatment of agricultural wastes

Wheat bran, date's seeds, grass and palm leaves were chosen as the sole nutrient source for solid-state fermentation (SSF). They dried in an oven at 80 °C for 24 h. The dried substrates were then milled in a commercial mill and sieved. The mean diameter of the dried substrates was 1.0 mm. The substrates were pretreated with 1.0 M NaOH at 121 °C and 15 psi pressure for 1 hr at the ratio of 1:10 (w/v) [23,24]. The pretreated materials were washed with tap water until the pH of the filtrate reached 7.0. The washed materials were dried at 60 °C overnight to constant weight and stored at room temperature for further use.

### Inoculum medium

The medium used for inoculum of *Trichoderma* sp. preparation contained (g l^−1^): KH_2_PO_4_, 28; (NH_4_)_2_SO_4_, 19.6; Urea, 4.2; MgSO_4_. 7H_2_0, 4.2; CoCl_2_, 4.2; FeSO_4_. 7H_2_0, 0.07; MnSO_4_. 7H_2_0, 0.021; ZnSO4 7H_2_0, 0.019; CaC1_2_, 0.028; yeast extract, 7; and glucose, 15; pH 5.0 ± 0.2. The media were sterilized by autoclaving at 121 °C pressure of 15 psi for 15 min. The culture was incubated and shaken at 30 °C for 48 hr in an orbital shaking incubator at 150 rpm before transferring to the production medium [25].

### Solid state fermentation

SSF was performed to study the effect of various physicochemical parameters required for the optimum production of enzymes and saccharification content by *Trichoderm* sp. prior to inoculation, the agriculture waste was sterilized in an autoclave for 20 min at 121 °C and 1.2 atmospheres. To each 50 ml Erlenmeyer flask, 5 g of sterilized agriculture waste, 5 × 10^5^ spores/g, and appropriate amount of water (10 % moisture) were added. The physico-chemical parameters included incubation time, incubation temperature (20, 30, 35, 40, 45 °C), moisture content of the substrate (10 %, 20 %, 40 %, 60 %, 100 %) and incubation pH (4 to 8) were optimized. The pH was adjusted using 0.1 M NaOH or HCl. Studies were also performed to evaluate the influence of different carbon sources (glucose, maltose, starch, sucrose, lactose at 1 % w/v) and nitrogen sources (yeast extract, urea, sodium nitrate, ammonium sulphate, ammonium chloride at 1 % w/v) when added to the fermentation medium contained agriculture waste. Each experiment is done in 3 sets.

### Enzyme extraction

Crude enzyme was extracted by mixing a 5 g of fermented matter with 50 ml distilled water on a rotary shaker (180 rpm/min) overnight. The suspension was then centrifuged at 12000 rpm for 10 min and the supernatant was designated as a crude extract.

### Enzyme assays

Carboxymethylcellulase (CMCase), filter paperase (FPase), pectinase (PGase) and xylanase (Xylase) activities were assayed by determining the liberated reducing end products using glucose, glucose, galacturonic acid and xylose as standards, respectively [26]. Substrates used were CM-cellulose, filter paper, polygalacturonic acid and birchwood xylan for CMCase, FPase, PGase and Xylase, respectively. The reaction mixture (0.5 ml) contained 1 % substrate, 0.05 M sodium acetate buffer pH 5.5 and 0.1 ml crude extract. Assays were carried out at 37 °C for 1 h. Then 0.5 ml dinitrosalicylic acid reagent was added to each tube. Then the reaction mixture was mixed well, and heated in a boiling water bath for 10 min. After cooling to room temperature, the absorbance was measured at 560 nm. One unit of enzyme activity is defined as the amount of enzyme which liberated one μmol of reducing sugar per min under standard assay conditions. All the experimental work was run in triplicates.

### Protein determination

Protein concentration was determined according to the dye binding method of Bradford [27] with bovine serum albumin as standard.

### Determination of total reducing sugars

Total reducing sugars were determined by the method of Miller [26]. The reaction mixture contained 0.5 ml of crude extract and 0.5 ml dinitrosalicylic acid reagent. The tubes were heated in a boiling water bath for 10 min. After cooling to room temperature, the absorbance was measured at 560 nm. Glucose served as the calibration standard for total reducing sugar determination.

### Determination of total soluble carbohydrates

Total soluble carbohydrates were determined by the method of Dubois et al. [28]. The reaction mixture contained 25 μl of a 4:1 mixture of phenol and water, 0.8 ml of crude extract and 2 ml of concentrated sulfuric acid. Then mixed well, and heated in a boiling water bath for 30 min. The absorbance was determined at 480 nm. Glucose served as the calibration standard for total carbohydrate determination.

### Statistical analysis

The obtained data were statistically analyzed as a randomized complete block design with three replicates by analysis of variance (ANOVA) using the statistical package software SAS (SAS Institute Inc., 2000, Cary, NC., USA). Comparisons between means were made by *F-*test and the least significant differences (LSD) at level *P =* 0.05. Correlations coefficient among the different parameters were also calculated by SAS.

## Results and discussion

**Fig. 1:**
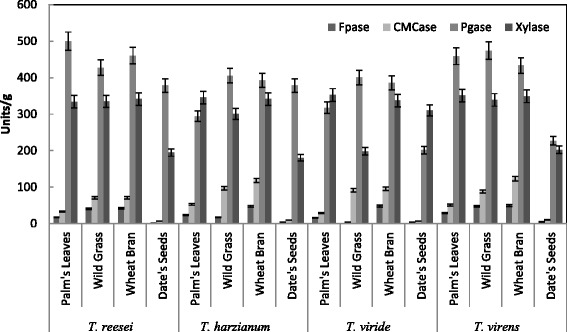
Effect of different organic materials on the production of CMCase, FPase, PGase and Xylase by *Trichoderma* sp. in SSF. Process conditions: incubation times 72 h, initial moisture content 50 % (by volume per mass) and temperature 30 °C. The data presented were averages of three experiments

**Fig. 2:**
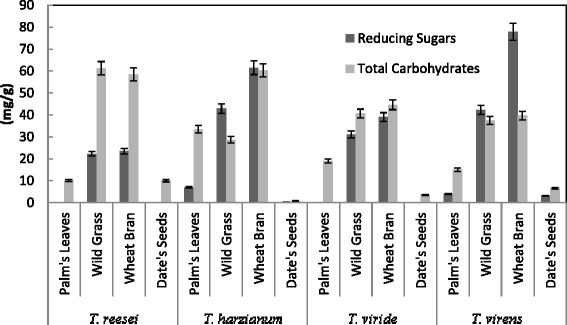
Effect of different organic materials on Saccharification content by *Trichoderma* sp. in SSF. Process conditions: incubation times 72 h, initial moisture content 50 % (by volume per mass) and temperature 30 °C. The data presented were averages of three experiments

Production of CMCase, FPase, PGase and Xylase were tested using alkali pretreated wheat bran, date's seeds, wild grass and palm leaves as substrates by *T. reesei, T. viride, T. harzianum* and *T. virens* in solid state fermentation (SSF). Figure 1 showed that the maximum production of CMCase, FPase, Xylase were obtained by *T. virens* (123.26, 49.3 and 348 U/g solid, respectively) in SSF containing alkali pre-treated wheat bran, while maximum PGase activity was obtained by *T. reesei* (499.9 U/g solid) in SSF containing alkali pre-treated palm leaves. Among the different solid substrates screened, saccharification of alkali pre-treated wheat bran supported maximum yields of total carbohydrates and reducing sugars (45 and 38.92 mg/g solid, respectively) by *T. virens* compared with other *Trichoderma* sp. tested (Fig. 2). There was no relation between total carbohydrate/sugar and enzyme contents, except of wild grasses and wheat bran. This may be attributed to some fungi used the total carbohydrate and sugar as nutrients (produced by enzymes act on certain agricultural residues as palm leaves and date seeds) for growth of fungi, resulted in the depletion of the total carbohydrate and sugar. From these results, the optimization of the production of total carbohydrate, reducing sugar, CMCase, FPase, PGase and Xylase of *T. virens* using alkali pre-treated wheat bran in SSF was performed in the following studies.

**Fig. 3:**
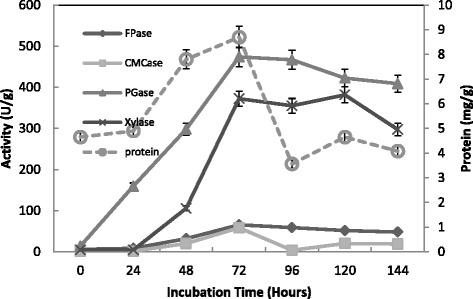
Effect of incubation time on the production of CMCase, FPase, PGase, Xylase and protein by *T. virens* in SSF using alkali pre-treated wheat bran as a substrate. Process conditions: incubation times 72 h, initial moisture content 50 % (by volume per mass) and temperature 30 °C. The data presented were averages of three experiments

Figure 3 shows the time course experiments of CMCase, FPase, Xylase, PGase and protein production by *T. virens* grown on alkali pretreated wheat bran in SSF. All enzymes and protein exhibited their maximum activities 58.2, 65.6, 372.4, 474 U/g and 8.5 mg/g at 72 hours. However, time course of enzyme cellulase production by *Trichoderma* spp. was studied using steamed alkali-treated sugarcane bagasse at 30 °C for six days. CMCase biosynthesis was not detected up to 24 hours of incubation and then the enzyme activity increased sharply up to 72 hours and highest CMCase activity was found 1.31 U/ml at 144 hours. FPase production started after a large lag period (about 72 hours) and thereafter the enzyme synthesis increase sharply. The final FPase activity was 0.110 U/ml at 144 hours. Initially, Xylase production was low and at 96 hours a sharp increase in enzyme production was observed and after that the enzyme activity remained constant. The highest Xylase activity was found after 144 hours of incubation which was 13.250 U/ml [29]. Of all four strains of fungi, *T. reesei* and *T. viride* were the best cellulase-producing fungi after 72 h offer mutation. A co-culture of *T. reesei* and *T. viride* gave better results with wheat straw; the CMCase activities were 560.9 and 212.7 IU, respectively [30]. Another study reported that PGase and Xylase of *T. virens*, exhibited their maximum activity at day 4 (132 and 75 units/g solid, respectively) [31]. Similarly, *Penicillium decumbens* was grown on a mixture of corn straw (90 %) and wheat bran (10 %) the maximum activity of Xylase was measured after 4 days of fermentation [32]. Couri et al. [33] also studied the production of Xylase by *A. niger* using different agroindustrial residues – mango peel and wheat bran – as the solid substrate. The maximum of xylase activity was reached after 72 h and 24 h of fermentation using wheat brain and mango peel, respectively.

**Fig. 4:**
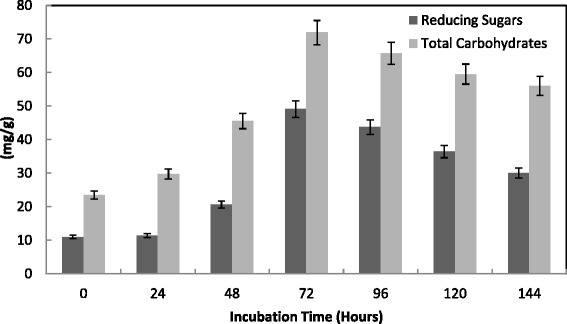
Effect of incubation time on the saccharification content by *T. virenss* in SSF using alkali pre-treated wheat bran. Process conditions: initial moisture content 50 % (by volume per mass) and temperature 30 °C. The data presented were averages of three experiments

The increase in glucose production depends on availability of cellulose in the medium and also due to the specific binding of the enzymes with substrates [34]. Saccharification of alkali pre-treated wheat bran in SSF was shown in Fig. 4 with a maximum yield 49.1 mg/g reducing sugars and 71.9 mg/g total carbohydrates obtained at 72 h by *T. virens*. Begum and Alimon [35] obtained the maximum amount of reducing sugar (4.15 mg/g) measured in sugarcane bagasse after alkali pre-treatment at 24 h by *Aspergillus oryzae* ITCC-4857.01. They also obtained the highest saccharification for alkali-reated sugarcane bagasse at 96 hrs when water hyacinth induced enzyme was used. They concluded that the saccharification rates of alkali pre-treated substrates were higher than those of enzymatic treated substrates. Similarly, Ja’afaru and Fagade [36] recorded the highest reducing sugar 5.55 mg/ml for alkali pre-treated corn cob at 48 hrs. Time course of enzymatic saccharification of alkali pre-treated bagasse showed rapid initial increase of reducing sugar concentration (up to 8 h) and the rate of this increase was substantially reduced at later stages by *T. viride* [37].

**Fig. 5:**
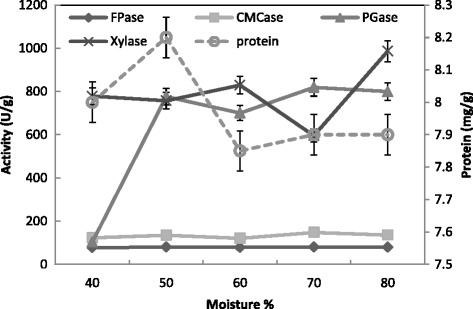
Effect of moisture % on the production of CMCase, FPase, PGase, Xylase and protein by *T. virens* in SSF using alkali pre-treated wheat bran as a substrate. Process conditions: incubation times 72 h, and temperature 30 °C. The data presented were averages of three experiments

**Fig. 6:**
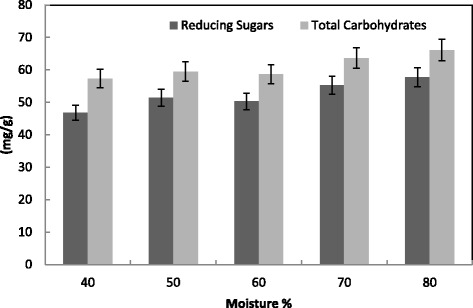
Effect of moisture % on the saccharification content by *T. virens* in SSF using alkali pre-treated wheat bran as substrate. Process conditions: incubation times 72 h and temperature 30 °C. The data presented were averages of three experiments

The moisture content was an important factor that influences the growth and product yield in SSF [38]. Moisture was reported to cause swelling of the substrates, thereby facilitating better utilization of the substrate by microorganisms [39,40]. The data presented in the Figs. 5 and 6, clearly indicated a maximum production of the enzymes CMCase, FPase, Xylase and PGase ranged from 50 to 70 % moisture. The highest level of protein production was detected at 50 % moisture. For CMCase production moisture level was optimized and found that 40 % moisture level was best for production by *T. viridei* [41]. Any further increase in the ratio resulted in the decrease of enzyme yields may be due to clumping of solid particles which results in the decrease of interparticle space leading to decreased diffusion of nutrients [40,42]. In contrast, the low moisture content leads to the decreased solubility of nutrients present in the wheat bran thereby decreases enzyme yields [43]. The optimum moisture contents for Xylase production by *T. longibrachiatum* and *A. tereus* were 55 and 75 %, respectively [44]. A high production of Xylase of *Aspergillus* species was detected at 40–50 % moisture using dry koji as substrate [45]. An initial moisture content of 40 % provided better conditions for production of PGase from *A. niger* than those of 25, 55, and 70 % [46].

**Fig. 7:**
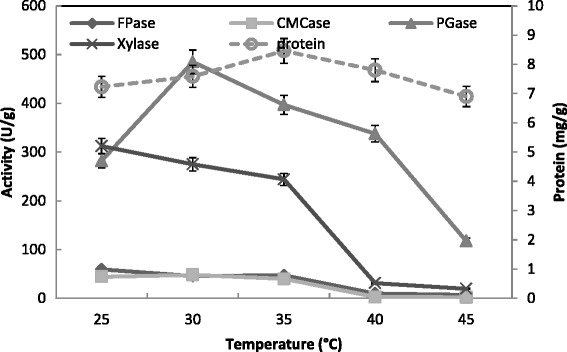
Effect of incubation temperature on the production of CMCase, FPase, PGase, Xylase and protein by *T. virens* in SSF using alkali pre-treated wheat bran as a substrate. Process conditions: incubation times 72 h and initial moisture content 50 % (by volume per mass). The data presented were averages of three experiments

**Fig. 8:**
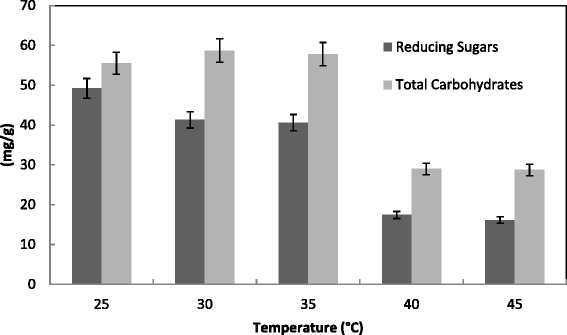
Effect of incubation temperature on the saccharification content by *T. virens* in SSF using alkali pre-treated wheat bran as substrate. Process conditions: incubation times 72 h and initial moisture content 50 % (by volume per mass).The data presented were averages of three experiments

The incubation temperature is very important in enzyme production [47]. The usual temperature maintained in SSF systems was in the range of 25-32 °C, depending on the growth kinetics of microorganism employed for fermentation purposes [48]. In the present study, the optimum temperature for maximum production of both CMCase and PGase was 30 °C; and 25 °C for FPase and xylase at 35 °C (Fig. 7). The protein content was ranged from 7 to 8 mg/g for all temperatures tested with high level at 35 °C. This was confirmed by maximum reducing sugar (49.2 mg/g) at 25 °C and total carbohydrates (55.5 mg/g) at 30 °C (Fig. 8). In contrast, maximum hydrolysis of substrates occurred at 50 °C [49]. Other studies had reported that 40 °C was found best for CMCase secretion by *T. viride* [41]. Optimal PGase and Xylase production (130 and 74 units/g solid, respectively) was obtained at 35 °C and 28 °C for *T. harzianum*, respectively [31]. On the contrary, the maximum activity of PGase and Xylase (120 and 55 units/g solid, respectively) of *T. virens* was detected at 28 °C and 35 °C, respectively. Similar optimal temperatures of production of PGase and xylanase from *Penicillium decumbens* [32], *A. niger* [33], *A. oryzae* [47], and *A. awamori* [50] were ranged from 28 °C to 32 °C. In the steady state operation for production of Xylase by *A. niger* the optimum temperature was 28 °C. Results showed that higher incubation temperature favors biomass growth and lower temperature favors the biosynthesis of xylanase [51]. The maximal PGase production by mixed culture of *A. niger* and *Saccharomyces cerevisiae* was detected at 37 °C [52].

**Fig. 9:**
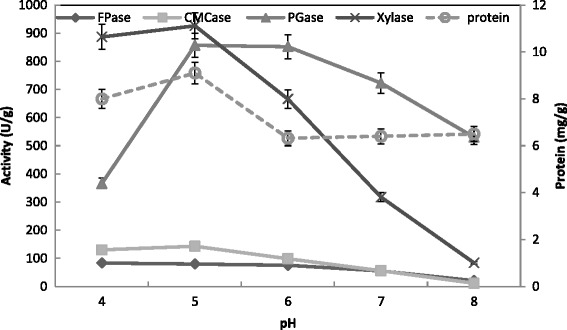
Effect of pH on the production of CMCase, FPase, PGase, Xylase and proein by *T. virens* in SSF using alkali pre-treated wheat bran as a substrate. Process conditions: incubation times 72 h, initial moisture content 50 % (by volume per mass) and temperature 30 °C. The data presented were averages of three experiments

**Fig. 10:**
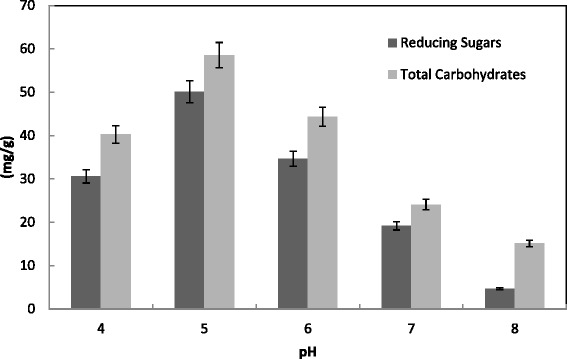
Effect of pH on the saccharification content by *T. virens* in SSF using alkali-pre-treated wheat bran. Process conditions: incubation times 72 h, initial moisture content 50 % (by volume per mass) and temperature 30 °C. The data presented were averages of three experiments

Figure 9 showed a maximum activity of CMCase (110 U/g), FPase (95 U/g), Xylase (910 U/g), PGase (820 U/g) and protein (9 mg/g) for *T. virens* at pH 5.0. This was reinforced by a maximum saccharification at the same pH 5 (Fig. 10). Another study reported a maximum degree of saccharification at pH 5.0 by *Trichoderma* sp. [29]. Maximum CMCase activity (16.2 U/ml) was also obtained at pH 5.5 by *T. viride* [41]. Optimal PGase and Xylase production of *T. harzianum* was obtained at pH 7 and 6 with 120 and 70 units/g solid, respectively. On the contrary, the maximum activity of PGase and xylanase of *T. virens* was detected at pH 6.0 and 7.0 with 140 and 60 units/g solid, respectively [31]. At the medium pH (6.0), the maximal xylanase production by *A. terreus* under SSF using palm as substrate was reported [53]. Patil and Dayanand [54] reported that pH 5.0 was optimum for the maximum production of pectinases of *A. niger* using deseeded sunflower head in both submerged and solid state fermentation.

**Fig. 11:**
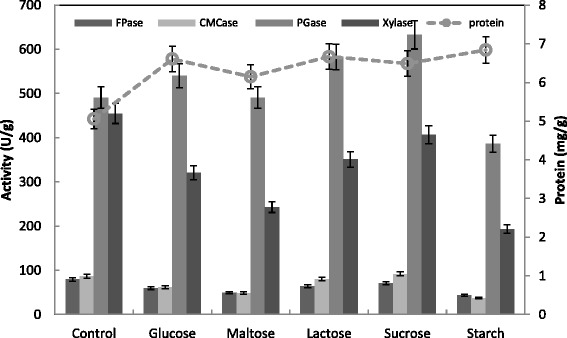
Effect of carbon source (1 %) supplementation on the production of CMCase, FPase, PGase, Xylase and protein by *T. virens* in SSF using alkali pre-treated wheat bran as a substrate. Process conditions: incubation times 72 hr, initial moisture content 50 % (by volume per mass) and temperature 30 °C. The data presented were averages of three experiments

**Fig. 12:**
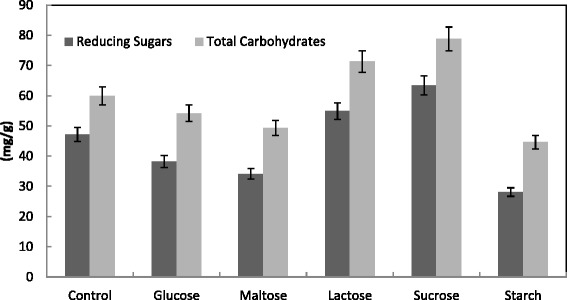
Effect of carbon source (1 %) supplementation on the saccharification content by *T. virens* in SSF using alkali pre-treated wheat bran. Process conditions: incubation times 72 h, initial moisture content 50 % (by volume per mass) and temperature 30 °C. The data presented were averages of three experiments

The influence of supplementary carbon sources such as starch, sucrose, maltose, lactose or glucose at 1 % (by mass) on production of CMCase, FPase, Xylase, PGase and protein was studied. The protein content was slightly increased in presences of the carbon sources. While lactose and sucrose enhanced the activity of PGase from 79.2 to 582.9 and 632.6 U/g solid, respectively, starch inhibited all other enzymes (Fig. 11). This was confirmed by maximum saccharification content of 54.9 and 63.4 mg/g reducing sugars respectively by lactose and sucrose, and minimum reducing sugars of 28.1 mg/g by starch (Fig. 12). However, starch and sucrose enhanced the Xylase activities from 40 to 55–60 units/g solid, while all carbon sources exhibited slightly effect on PGase activities for *Trichoderma* spp. using cantaloupe and watermelon rinds [31]. Botella et al. [50] reported that when 6 % glucose was added as extra carbon source, the production of Xylase and PGase by *A. awamori* using grape pomace as substrate increased significantly. However, at 8 % both enzyme activities declined. In contrast, when wheat bran was used as the solid substrate, Xylase by *A. tamari* was resistant to catabolic repression even at 10 % glucose [55]. Alternatively, D-glucose, D-mannose, maltose, sucrose and cellobiose significantly repressed CMCase formation in case of using rice straw and sugar cane bagasse as carbon sources [56].

**Fig. 13:**
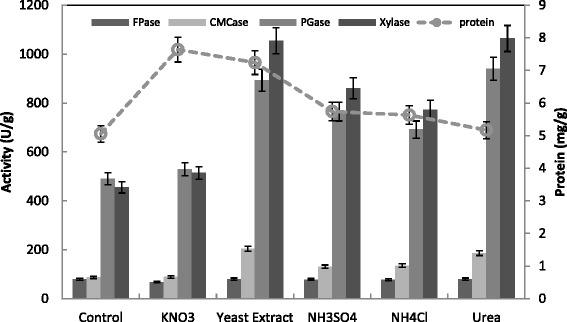
Effect of nitrogen source (1 %) supplementation on the production of CMCase, FPase, PGase, Xylase and protein by *T. virens* in SSF using alkali pre-treated wheat bran as a substrate. Process conditions: incubation times 72 h, initial moisture content 50 % (by volume per mass) and temperature 30 °C. The data presented were averages of three experiments

**Fig. 14:**
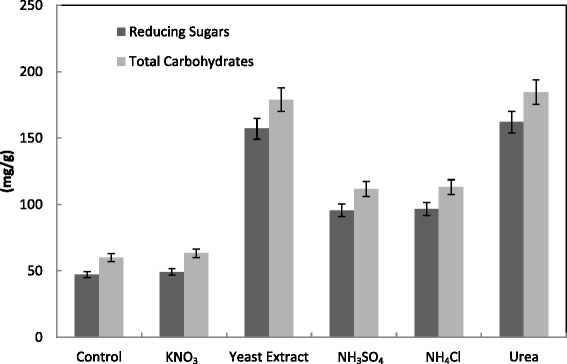
Effect of nitrogen source (1 %) supplementation on the saccharification content by *T. virens* in SSF using alkali-pre-treated wheat bran. Process conditions: incubation times 72 h, initial moisture content 50 % (by volume per mass) and temperature 30 °C. The data presented were averages of three experiments

Studies on supplementation of nitrogen sources such as ammonium sulphate, ammonium nitrate, ammonium chloride, yeast extract or urea at 1 % concentration to the solid substrates showed various effects on CMCase, FPase, Xylase, PGase and protein production by *T. virens* (Fig. 13). All nitrogen sources enhanced the saccharification enzymes with significant enhancement for CMCase (from 86.9 to 204 U/g), PGase (from 491.1 to 939.4 U/g) and Xylase (from 455 to 1064 U/g) by yeast extract and urea. This was confirmed by maximum saccharification from 47.2 to 157.1 and 162.1 by yeast extract and urea respectively (Fig. 14). However, ammonium sulphate, ammonium nitrate, yeast extract and urea increased PGase activities of *T. harzianum* from 90 to 110–113 units/g solid and decreased PGase activities of *T. virens.* The high level of protein was detected in presence of KNO_3_. However, urea increased Xylase activities of *T. harzianum* and *T. virens* [30]*.* Among nitrogen sources yeast extract was the best to enhance the enzyme activity FPase (0.281 ± 0.13 IU/g) and CMCase (3.66 ± 0.02 IU/g) by *A. fumigatus* grown on alkali-pretreated sawdust [57]. Another study reported the highest level of enzyme formation expressed in terms of specific activity with ammonium chloride with both rice straw and sugar cane bagasse by *A. terreus* DSM 826. Other nitrogen sources namely ammonium sulphate, potassium nitrate and ammonium oxalate gave also considerable amounts of CMCase with both wastes as compared with sodium nitrate [56].

Comparison of CMCase, FPase, PGase and xylase production pattern by *T. virens* used in this study and other microorganisms confirmed the potential of *T. virens* for economic hydrolytic enzymes production (Table 1). In fact, the present microorganism was better producer of FPase, CMCase, Xylase and PGase (approximately 49.3, 123.26, 348 and 499.9 units/g solid, respectively) compared to other microorganisms.

**Table 1:** Sacchrification enzyme activities of different fungal isolates grown on lignocellulosic substrates under solid state fermentation

**Enzyme source**	**Carbon source**	**Enzyme activities (U/g dry substrate)**				**References**
		**FPase**	**CMCase**	**Xylase**	**PGase**	
*Aspergillus ustus*	Rice straw	5.82	12.58	740	-	Shamala and Sreekantiah [58]
	Wheat bran	3.78	11.84	615.26	-	
*Aspergillus soja*	Crushed maize	-	-	-	30	Ustok et al. [59]
*Aspergillus awamori*	Grape pomace	-	-	40	25	Botella et al. [60]
*Aspergillus terreus* M11	Corn stover	243	581	-	-	Gao et al. [61]
*Aspergillus niger* KK2	Rice straw	19.5	129	5070	-	Kang et al. [62]
*Aspergillus niger*	Ordos Plateau	-	-	-	36	Debing et al. [63]
*Aspergillus niger*	Citrus peel	-	-	65	18	Rodriguez-fernandez et al. [64]
*Aspergillus niger*	Deseeded sunflower	-	-	-	34	Patil and Dayanand [54]
*Myceliophthora* sp. IMI 387099	Rice straw	2.44	32.9	900.2	-	Badhan et al. [65]
	Wheat straw	1.37	30.8	656.6	-	
	Bagasse	0.7	6.62	620.1	-	
	Corn cob	0.31	11.38	411.6	-	
	Wheat bran	0.74	26.6	128.9	-	
*Thermoascus aurantiacus*	Wheat straw	4.3	956	2973	-	Kalogeris et al. [66]
*Trichoderma harzianum* and *Trichoderma virens*	cantaloupe and watermelon	-	-	80	140	Mohamed et al. [31]
*Trichoderma harzianum* SNRS3	Rice straw	6.25	111.31	433.75	-	Rahnama et al. [67]
*Trichoderma reesei* MCG77	Rice bran	2.314	-	-	-	Latifan et al. [68]
*Trichoderma virens*	Wheat bran	49.3	123.26	348	499.9	Present study

## Conclusion

The present study revealed the saccharification potential of *T. virens* on alkali pre-treated wheat bran as an agricultural waste in SSF. The optimal conditions for production of CMCase, FPase, PGase and xylase and sccharification content utilizing alkali pre-treated wheat bran as the solid substrate in SSF included incubation for 72 h, temperature at 25-35 °C, substrate moisture content of 50-70 % and pH at 5.0.
